# Interleukin-34 Mediates Cross-Talk Between Stromal Cells and Immune Cells in the Gut

**DOI:** 10.3389/fimmu.2022.873332

**Published:** 2022-04-22

**Authors:** Giovanni Monteleone, Eleonora Franzè, Edoardo Troncone, Claudia Maresca, Irene Marafini

**Affiliations:** ^1^ Department of Systems Medicine, University of Rome Tor Vergata, Rome, Italy; ^2^ *Gastroenterology Unit, Policlinico Tor Vergata, Rome, Italy

**Keywords:** cytokines, Crohn’s disease, ulcerative colitis, mucosal inflammation, intestinal fibrosis, M-CSF1

## Abstract

Initially known as a cytokine produced by and regulating the function of monocytes and macrophages, interleukin-34 (IL-34) can be synthesized by many cell types and interacts with receptors expressed by multiple immune and non-immune cells. IL-34 is constitutively expressed in the healthy human small intestine and colon and its production is markedly increased in damaged gut of patients with Crohn’s disease and patients with ulcerative colitis, the main forms of chronic inflammatory bowel diseases (IBD) in human beings. Circumstantial evidence suggests that, in these pathologies, IL-34 plays a crucial role in mediating cross-talk between immune cells and stromal cells, thereby promoting activation of signalling pathways, which amplify the ongoing mucosal inflammation as well as production of fibrogenic molecules. In this article, we summarize the available data supporting the multiple effects of IL-34 in human IBD with particular attention to the role of the cytokine in immune and stromal cell interactions.

## Introduction

Crohn’s disease (CD) and ulcerative colitis (UC) are the main forms of chronic inflammatory bowel diseases (IBD) in human beings (1–3). These pathologies are characterized by various degrees of mucosal damage and tissue remodelling, which favour the development of ulcers, strictures and fistulae, and local complications (1, 2). The aetiology of both CD and UC remains unknown, but a large body of evidence suggests that both diseases are due to interaction among multiple genetic, environmental and microbial factors, which eventually triggers and sustains an excessive immune reaction within the intestinal wall direct against components of the luminal flora (4, 5).

In inflamed gut of IBD patients, there is massive presence of immune cells (e.g. neutrophils, T and B lymphocytes, natural killer (T) cells and CD14+ monocytes), which are continuously recruited from the peripheral blood (6). These cells synthesize huge amounts of cytokines that can trigger inflammatory signals in both immune and non-immune cells, thus contributing to expand the pathologic process (7). Consistent with this is the demonstration that blockade of effector cytokines, such as interleukin (IL)-12/IL-23 and tumor necrosis factor (TNF), helps attenuate the detrimental responses in the gut of IBD patients (8–10). However, some patients are primarily or secondarily resistant to such drugs or develop drug-related adverse events, thus suggesting the necessity of additional therapeutic agents (11).

Initially known as a cytokine produced by monocytes and macrophages, IL-34 can be synthesized by additional cell types, including epithelial cells, macrophages, endothelial cells, fibroblasts, neurons, hepatocytes and, and is constitutively expressed in many adult human organs, such as heart, brain, liver, spleen, thymus, testis, ovary, prostate, small intestine, and colon (12–16).

IL-34 is a powerful regulator of myeloid cell differentiation and behaviour. In addition, IL-34 mediates the function of many other cell types with the downstream effect of either amplifying or limiting immune-inflammatory responses in some organs/tissues (17–24).

In this article, we review the available data supporting the multiple effects of IL-34 in human IBD with particular attention to the role of the cytokine in promoting stromal and immune cell interactions. We discuss also similarities and differences in the roles of IL-34 and macrophage colony-stimulating factor (M-CSF-1) in the control of gut homeostasis and inflammation as these two molecules bind the same receptor, namely macrophage colony-stimulating factor receptor (M-CSF-1R) (12).

## Interleukin 34 Signalling

Although IL‐34 and M-CSF‐1 bind to overlapping regions of M-CSF-1R (25) they can activate distinct signalling pathways depending on the hydrophobic/hydrophilic interaction of each cytokine with M-CSF1-R (26–30). IL-34 binds to M-CSF-1-R by hydrophobic interaction while M-CSF-1 binding to M-CSF-1R is mediated by a hydrophilic interaction. Hydrophobic interactions are more stable than hydrophilic ones and, therefore, IL-34 induces a stronger and faster auto-phosphorylation of tyrosine residues of the intracellular domain of M-CSF-1R, as well as of FAK, MAP Kinases and other downstream targets as compared to M-CSF-1 (30). In human monocytes, IL-34 and M-CSF-1 are equally able to promote phosphorylation of AKT, ERK1/2, AMPK and ULK1 (31, 32). These differences in intracellular pathways help explain the non-redundant roles of the two cytokines on some macrophage functions, as well as the fact that IL-34 activity on monocytes is independent of M-CSF-1 (12). In this context, for instance, it has been demonstrated that IL-34-differentiated macrophages exhibit over-expression of eotaxin 2 and HLA-DR and down-regulation of CD54 and MCP-1 as compared to M-CSF-1-stimulated macrophages (30). Moreover, M-CSF-1-treated macrophages have enhanced capacity for bacteria phagocytosis as compared to IL-34-derived macrophages (33). IL-34-derived macrophages and M-CSF-1-derived macrophages produce similar amount of IL-10 but low level of IL-12 (34). However, upon inflammatory macrophage type 1 (M1) polarization (i.e. LPS + IFNγ), IL-34-treated macrophages express higher IL-10 and CXCL11 levels than M-CSF-1-stimulated macrophages, while in response to regulatory M2 polarization (i.e. IL-4), IL-34-treated macrophages produce more chemokine ligand (CCL) 17 and CCL22 and less IL-10 than M-CSF-1-differentiated macrophages (31).

Unlike M-CSF-1, which only interacts with M-CSF-1R, IL-34 can bind to additional receptors, such as receptor-type protein-tyrosine phosphatase zeta (PTP-ζ), a chondroitin sulfate proteoglycan primarily expressed on neuronal progenitors and glial cells, kidney tubular cells, monocytes and B cells, and syndecan-1 (also known as CD138), a heparane sulfate and/or chondroitin sulfate proteoglycan prevalently expressed by epithelial cells and able to bind many other ligands (i.e. proteins of the extracellular matrix, cytokines and growth factors (30, 31).

During embryogenesis as well as at the adult stage, IL-34 and M-CSF-1 have distinct expression patterns and there is evidence that M-CSF-1 exerts a more systemic function while IL-34 has tissue-restricted actions. Studies in knockout mice showed that loss of M-CSF-1R or M-CSF-1 led to a more severe phenotype, with severe deficiency in mature macrophages and osteoclasts, reduced lifespan, growth, fertility as compared to IL-34-deficient animals, probably reflecting the major involvement of M-CSF-1 in the development of osteoclasts, myeloid cells and in the production of IGF-1, the activity of which is essential to growth and organ maturity (14, 15, 35–38). However, M-CSF-1R-deficient animals exhibit a more harmful phenotype than M-CSF-1-deficient mice, indicating a contribution of IL-34 in such mechanisms.

## Role of M-CSF-1R-driven Signalling in the Control of Gut Homeostasis

Analysis of M-CSF-1R in the gut revealed its expression throughout the crypts in both the mouse and human colon, to the crypt base (i.e. Paneth cells) of the small intestine as well as on lamina propria myeloid cells and stromal cells (21, 39, 40). However, data from Sehgal and colleagues contradicted this view and showed that M-CSF-1R mRNA expression is restricted to macrophages, which are intimately associated with the crypt epithelium, and is undetectable in Paneth cells (41). The reasons for the discrepancy between these studies are uncertain. Regardless of whether Paneth cells express M-CSF-1R, studies in mice lacking either M-CSF-1R- or M-CSF-1 clearly indicate a prominent role of M-CSF-1R in the control of epithelial cell differentiation. Specifically, M-CSF-1R loss impeded the development of M cells in Peyer’s patches, and associated with deficits in small intestinal enterocytes and enteroendocrine cells, excessive goblet cell production, and reduced crypt proliferative capacity and stem cell niche maintenance in the small intestine (42). Consistently, loss of either M-CSF-1R or M-CSF-1 resulted in defects in enterocytes and enteroendocrine cell fate, with excessive goblet cell production and reduced cell proliferation in the colon (39). These data are in line with the *in vitro* demonstration that M-CSF-1 enhances murine fetal and prenatal colon cell proliferation (40).

In order to assess the M-CSF1- and IL-34-mediated regulation of macrophage homeostasis, Lin and colleagues treated C57BL6 mice with highly selective antibodies against M-CSF-1 or IL-34, either separately or in combination for 4 weeks, and then evaluated various tissues by immunostaining using F4/80 antibody to identify resident macrophages. Treatment with anti-M-CSF-1 or anti-M-CSF-1+anti-IL-34 but not with anti-IL-34 alone reduced the number of intestinal macrophages. Although these findings do not help ascertain whether the effect of the IL-34/M-CSF-1 blockers on intestinal macrophage survival is either direct or indirect, they clearly indicate a dominant role of M-CSF-1 versus IL-34 in the control of macrophage homeostasis in the gut (43). Expression of M-CSF-1R on macrophages seems to be also crucial in the cross-talk between macrophages and intestinal epithelial cells. Indeed, it has been demonstrated that intestinal crypt-associated macrophages are required to maintain the crypts in the small intestine as depletion of macrophages by prolonged M-CSF-1R blockade inhibited Paneth cell differentiation and led to a reduction of Lgr5+ intestinal stem cells (41). The factors produced by the crypt-associated M-CSF-1R-expressing macrophages, which support Paneth cell differentiation, remain unknown.

We and others have shown that human intestinal stromal cells express M-CSF-1R and produce both M-CSF-1 and IL-34 (18, 44). By using a 3D human organotypic colorectal cancer model, Stadler and colleagues showed that stromal cell-derived M-CSF-1 can target M-CSF-1R on myeloid cells and positively regulate the macrophage number in the cultures as well as expression of chemokines and CD163 (44). The phenotype of M-CSF-1-stimulated macrophages resembles that of normal intestinal resident macrophages, which are highly positive for CD163, CD163L1 and CD206 and have the ability to promote epithelial barrier integrity and activate counter-regulatory pathways (45, 46). Overall, these findings support the view that M-CSF-1R signalling, by promoting the link between macrophages and epithelial cells/stromal cells, contributes to maintain gut homeostasis.

## IL-34 is Over-Expressed in the Gut of IBD Patients and Regulates Inflammatory Cytokine and Chemokine Production

As pointed-out above, IBD tissue is massively infiltrated with activated monocytes/macrophages and dendritic cells, which have the ability to respond to bacterial products by producing a large array of inflammatory molecules and tissue-degrading enzymes (4). Therefore, identifying the key regulators of the function of such cells could help advance our understanding about the basic mechanisms underlying the IBD-associated pathogenic process. Analysis of IL-34 RNA and protein expression in paired biopsy samples taken from involved and uninvolved mucosal areas of IBD patients and normal controls showed the predominant induction of the cytokine in inflamed gut of both CD patients and UC patients as compared to uninflamed gut of the same patients and healthy intestine (23). Accordingly, data from the IBD Transcriptome and Metatranscriptome Meta-Analysis (TaMMA) framework, a comprehensive survey of publicly available IBD RNA-sequencing datasets, confirmed the increased IL-34 gene expression in ileal CD compared to controls (47). Similarly, M-CSF-1 RNA transcripts were found to be elevated in inflamed gut of CD patients and UC patients compared to non-IBD control diverticulitis and normal tissues (43). Additionally, M-CSF-1 gene sets were enriched in the colonic mucosal transcriptomes of CD patients and particularly in those who did not respond to TNF blockers (48).

Up-regulation of both IL-34 and M-CSF-1 was documented in the colons of mice with dextran sulfate sodium (DSS)-colitis (49). Analysis of these two cytokines in gut samples of TNFΔ^ARE^ mice, which show some immunological similarities with CD (50), showed that M-CSF1 was increased in the ileum and cecum, whereas IL34 was elevated primarily in the jejunum and ileum (43).

**Figure 1:**
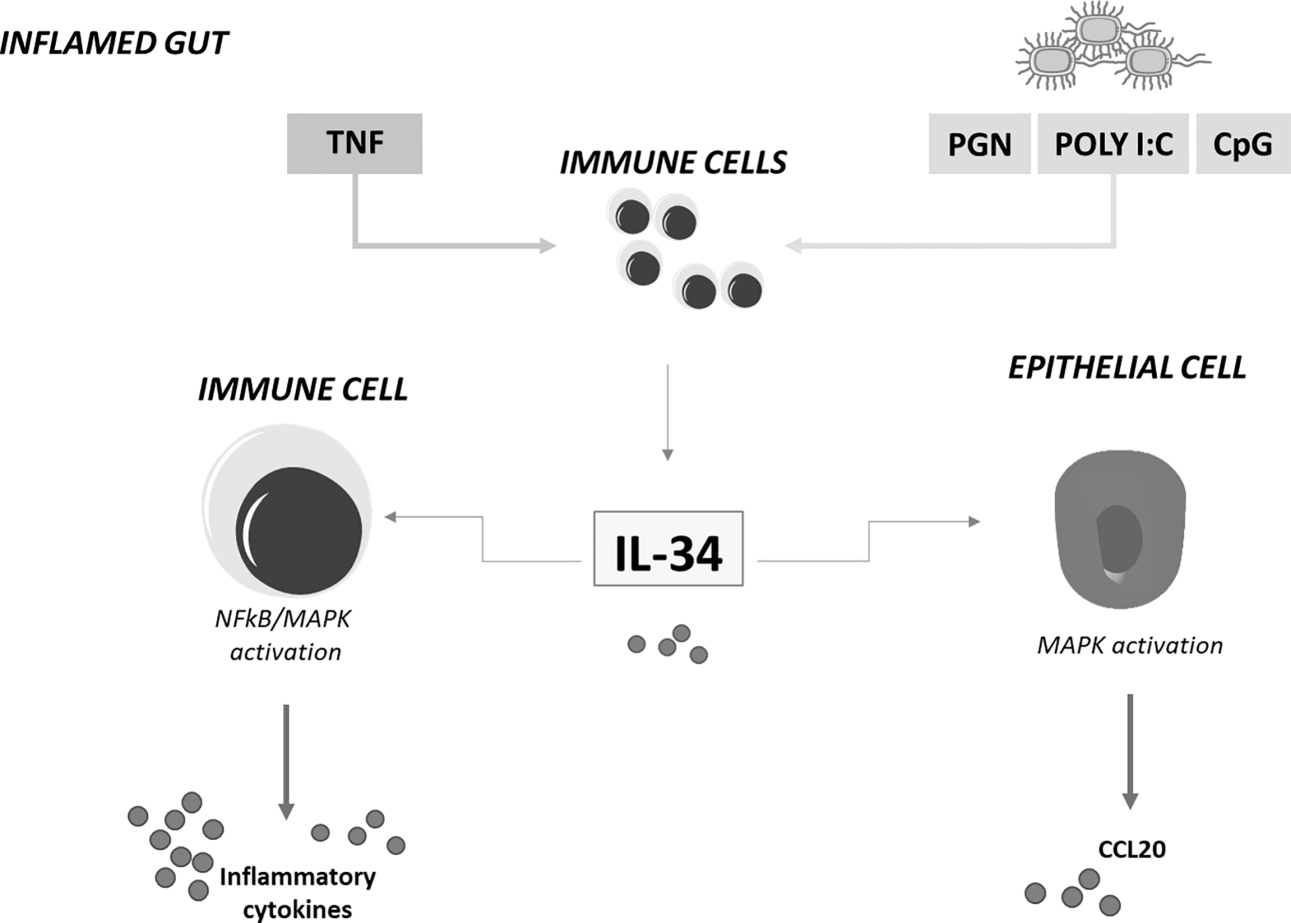
IL-34 regulates inflammatory cytokine and chemokine production in inflamed gut. IL-34 produced by lamina propria immune-inflammatory cells in response to TNF and toll-like-receptor stimulation stimulates immune cells to produce inflammatory cytokines and epithelial cells to secrete chemoattractants. MAPK, mitogen-activated protein kinase; NFkB, nuclear factor kappa-light-chain-enhancer of activated B cells; CCL20, chemokine (C-C motif) ligand 20; TNF, tumor necrosis factor; IL-34, interleukin-34; PGN, Peptidoglycan; CpG, 5'C-phosphate-G3'; Poly I:C, polyinosinic:polycytidylic acid.

Immunohistochemical analysis of human gut mucosal samples showed that, in IBD, IL-34 was over-produced not only by lamina propria myeloid cells but also by stromal cells and epithelial cells (23). These findings raised the possibility that, in IBD, IL-34 production is positively regulated by molecules synthesized and/or acting within the inflamed gut. Indeed, stimulation of unfractioned lamina propria mononuclear cells (LPMC) isolated from normal colonic samples with TNF, a cytokine over-produced in both CD and UC (4), increased IL-34 production while blockade of TNF with a neutralizing TNF antibody in IBD LPMC cultures and ex vivo mucosal explants taken from IBD patients reduced IL-34 synthesis. Additionally, several toll-like receptor ligands, such as peptidoglycan, Poly (I:C) and CpG stimulated IL-34 production in LPMC (23) (**Figure 1**). Functionally, IL-34 triggered ERK1/2 activation in normal colonic LPMC thus enhancing IL-6 and TNF production. Consistently, blockade of IL-34 with a neutralizing antibody reduced TNF and IL-6 expression in IBD mucosal explants. Overall, these findings fit with the demonstration that IL-34 has significant action in regulating immune responses through a complex signaling network, involving not only MAP kinases but also nuclear factor-kappa B, a crucial molecule for activation of inflammatory signaling processes (23) (**Figure 1**). Indeed, IL-34 has been reported to amplify production of pro-inflammatory cytokines, chemokines, and tissue-damaging proteases by various immune and non-immune cell types (51) and up-regulation of the cytokine has been seen in pathologies characterized by accumulation of pro-inflammatory monocytes in inflamed sites (52).

Studies in other systems have also shown that, under specific circumstances, IL-34 can act as a regulatory molecule and this relies in part on the ability of the cytokine to induce differentiation of monocytes to type 2 macrophages, which possess an immunosuppressive effect on NK and T cell responses (34, 53). However, treatment of intestinal LPMC with IL-34 did not change expression of CD206 and Arginase1, two markers of type 2 macrophages, thus suggesting that IL-34-driven induction of inflammatory cytokines in the gut is not secondary to shifts in the differentiation/development of monocyte/macrophages (23).

Although no study has yet evaluated the action of IL-34 on the differentiation/function of T cells in IBD, it is noteworthy that IL-34-primed macrophages constitutively express membrane-type IL-1α, which stimulates differentiation of memory Th17 cells (54), a subset of CD4+ T cells that infiltrates massively inflamed gut in IBD and contributes to mucosal injury seen in mice with experimental colitis (55, 56).

In IBD, the expansion of the pathogenic inflammatory response is partly mediated by recruitment of immune cells from the circulation to inflamed mucosa, as a result of interaction between integrins and adhesion molecules and action of multiple chemokines. Chemokines are mainly secreted by epithelial cells in response to inflammatory stimuli (e.g. cytokines) derived from immune cells and stromal cells (6, 57). IL-34 could be one these cytokines, as recent studies indicate that gut epithelial cells are a potential target of IL-34. Indeed, by immunohistochemistry, we showed that in the normal colon, epithelial cells constitutively expressed M-CSFR-1 even though M-CSFR-1-producing cells were more frequent in both the epithelial and lamina propria compartments of IBD patients (23). Stimulation of gut epithelial cells with IL-34, but not M-CSF-1, enhanced secretion of the CCL20 (21). Consistently, neutralization of IL-34 in ex vivo mucosal explants taken from IBD patients with a blocking IL-34 reduced CCL20 secretion (**Figure 1**). These findings could be pathogenetically relevant as CCL20 is highly produced by IBD epithelial cells and supposed to contribute to the recruitment of immune cells from the circulation to inflamed intestine (58).

Functional studies aimed at evaluating the effect of M-CSF-1 and/or IL-34 neutralization in murine models of colitis showed that pre-treatment of mice with both anti-M-CSF1 and anti-IL-34 one day prior to DSS challenge reduced the number of intestinal macrophages, the expression of inflammatory cytokines and limited damage as compared to mice treated with control antibody. Single treatment with either anti-M-CSF1 or anti-IL-34 was only marginally beneficial. Similarly, dual inhibition of IL-34 and M-CSF-1 improved body weight and reduced gut inflammation in TNFΔ^ARE^ mice while single monotherapies were not effective. Finally, simultaneous blockade of IL-34 and M-SCF-1 reduced intestinal inflammation developing in mice with IL-10 deficiency (43). Consistently, mice lacking M-CSF-1R were protected from DSS-induced colitis as compared to wild-type mice (39). Administration of JNJ-40346527, a small molecule inhibitor of M-CSF-1R, to mice with T-cell transfer colitis attenuated clinical disease scores and reduced inflammatory gene expression (48).

Altogether, these observations are consistent with the pathologic role of IL-34 and M-CSF-1 in the gut and indicate that neutralization of both IL-34 and M-CSF-1 can help dampen the local tissue-damaging inflammatory response.

## Interleukin-34 Enhances the Pro-fibrogenic Properties of Gut Stromal Cells

IBD course can be complicated by fibrosis and development of strictures. These complications are seen more frequently in patients with ileal CD, but fibrosis can be also found in colonic CD and UC (59, 60). The basic mechanisms underlying fibrosis are not yet fully understood, even though accumulating evidence supports the hypothesis that fibrosis results from the chronic activity of immune-inflammatory cells, which stimulate excessive deposition of extracellular matrix (ECM) proteins by stromal cells (e.g. fibroblasts) (61, 62). The activated stromal cells secrete in turn several pro-fibrogenic cytokines [e.g. TGF-β, interleukin (IL)-1β, and IL-6], with the downstream effect of enhancing ECM deposition and amplifying the fibrotic process (59, 63–66). Indeed, administration of compounds inhibiting the function of such profibrotic cytokines to mice prevented or cured colitis-induced intestinal fibrosis (67–71).

Since IL-34 has been involved in the control of lung and joint fibroblasts (72, 73), we examined whether IL-34 regulates gut stromal cell function. The initial studies showed that M-CSFR-1 expression was markedly increased in gut samples taken from CD patients with fibrostrictures as compared to samples taken from CD patients with inflammatory phenotype and normal controls and immunohistochemical analysis of such samples showed that stromal cells were highly positive for the receptor (18). We also showed that stromal cells isolated from fibrostricturing specimens of CD patients and expressing typical markers of myofibroblasts, such as vimentin, α-SMA, and CD90, responded *in vitro* to IL-34 by enhancing COL1A1 and COL3A1 expression and secretion of soluble forms of collagen. The positive effect of IL-34 on collagen synthesis was abrogated by a pharmacological inhibitor of p38 MAP kinase. In line with this was also the demonstration that IL-34 RNA and protein expression was up-regulated in fibrotic samples of CD patients as compared to both inflamed and normal controls and, in fibrostricturing CD tissues, stromal cells were largely positive for IL-34. Knockdown of IL-34 with a specific antisense oligonucleotide in CD myofibroblasts isolated from fibrostricturing specimens reduced both p38 MAP kinase activation, COL1A1 and COL3A1 expression, and secretion of collagen in the culture supernatants (18). It is thus plausible that intestinal myofibroblasts are both a cell source and a target of IL-34. This fits also with recent studies showing that IL-34 stimulates migration and proliferation of synovial fibroblasts isolated from patients with rheumatoid arthritis (74, 75) and enhances production of inflammatory cytokines by lung fibroblasts (72). The potential involvement of IL-34 in the amplification of fibrotic processes is supported by studies in other disorders. For example, IL-34 stimulates liver macrophages to acquire profibrotic propertiesy, and in chronically hepatitis C virus (HCV)-infected patients, serum level of IL-34 well correlate with the fibrosis stage (16). IL-34 is highly produced in patients with diffuse cutaneous systemic sclerosis, and the serum levels of the cytokine in such patients correlate positively with fibrotic score on chest computed tomography (76). Finally, in patients with non-alcoholic fatty liver disease, serum IL-34 level increases with the progression of fibrosis (77).

## Discussion

**Figure 2:**
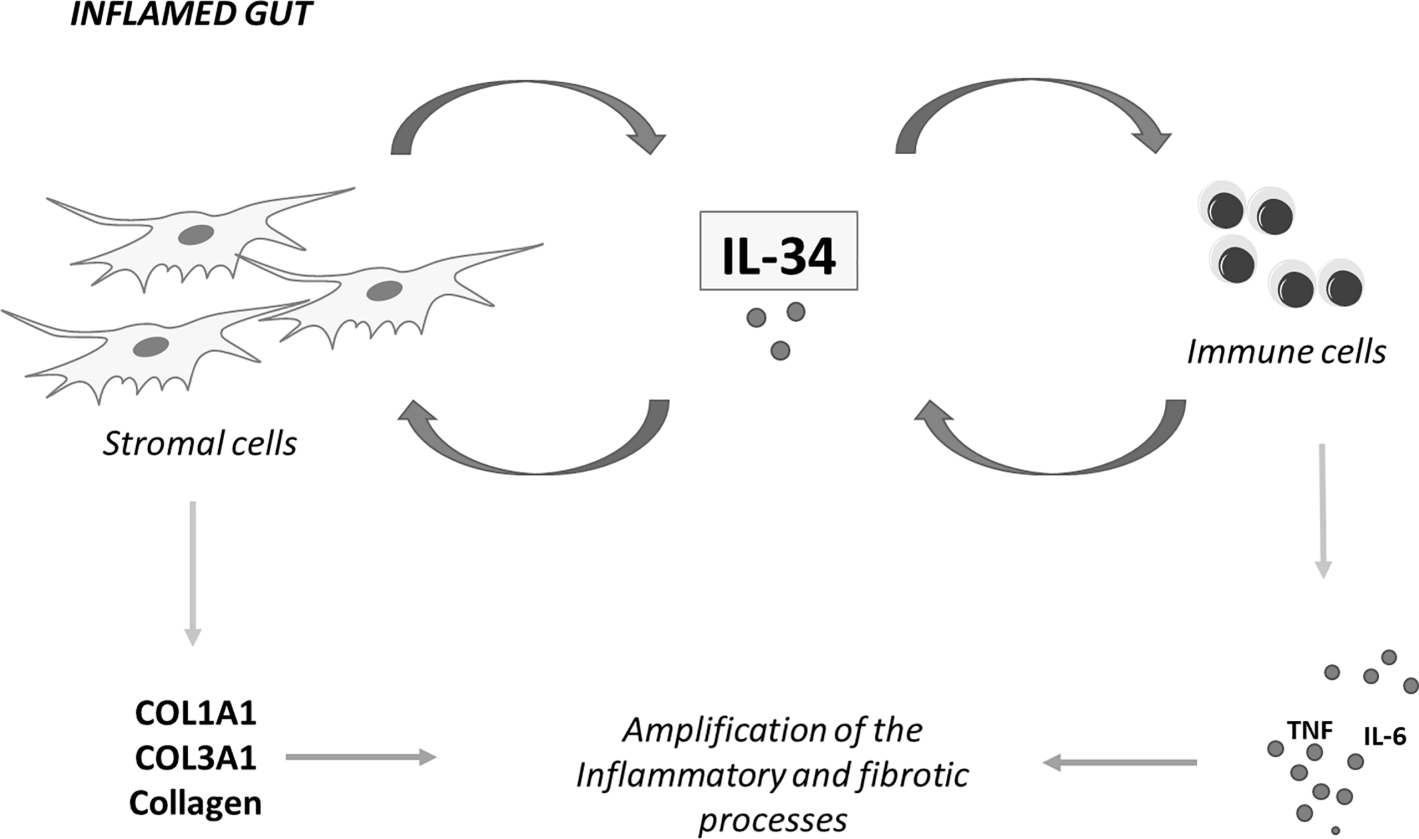
IL-34 mediates cross-talk between stromal cells and immune cells in inflammatory bowel diseases. IL-34 produced by intestinal stromal cells stimulates immune cells to make inflammatory cytokines and regulates, in an autocrine or paracrine manner, production of collagen by stromal cells. Similarly, immune cell-derived IL-34 regulates the function of both stromal cells and immune cells. COL1A1, collagen type I alpha 1 chain; COL3A1, collagen type III alpha 1 chain; TNF, tumor necrosis factor; IL-6, interleukin-6; IL-34, interleukin-34.

Our understanding about the involvement of IL-34 in IBD pathogenesis is still limited, but in recent years, some studies have highlighted the multiple effects of the cytokine on the ongoing mucosal inflammation in these disorders. It is now evident that IL-34 has many cell sources in the gut and signalling pathways activated by locally-produced cytokines and/or bacterial products sustain IL-34 production. At the same time, the main IL-34 receptor, namely M-CSFR-1, is simultaneously expressed by immune cells and non-immune cells, such as epithelial cells and stromal cells (18, 23). This helps delineate a scenario, in which IL-34, by acting *via* a paracrine or autocrine manner, either alone or in combination with M-CSF-1, can mediate the cross-talk among different mucosal cell types in IBD (**Figure 2**). Considering that IL-34 protein expression is barely detectable in the human healthy gut and its production is pronounced in inflamed gut of both CD patients and UC patients, it is conceivable that IL-34 may have a role in promoting tissue destruction. This hypothesis is supported by *in vitro* observations indicating that IL-34 triggers signals that, in the gut, amplify production of inflammatory cytokines and chemokine and pro-fibrogenic molecules (18, 21, 23). Nonetheless, very little is known about the *in vivo* function of the cytokine. Recent studies in wild-type mice with colitis treated with neutralizing antibodies indicate that depending on the disease, single or dual blockade of M-CSF-1 and IL-34 is needed for inhibiting mucosal inflammation consistent with the concordant tissue-specific expression of both cytokines in inflamed gut (43). Nonetheless, further studies are necessary to explore the role of both IL-34 and M-CSF-1 in additional models of colitis and colitis-associated fibrosis as we cannot exclude the possibility that selective inhibition of IL-34 may either not be adequate to control gut pathology or paradoxically exacerbate the disease. Indeed, it has been reported that IL-34 not only induces immunosuppressive macrophages but also enhances *in vitro* suppressive function of CD4+ and CD8+ regulatory T cells (Tregs) (61). IL-34 can be secreted by FOXP3-expressing CD4+ and CD8+ Tregs in humans and CD8+CD45RClow/- Tregs in rats (61) and, at least *in vitro*, it is more efficient at inducing FOXP3-expressing Tregs than M-CSF-1 and these cells efficiently control acute graft-vs.-host disease *in vivo* in a model of immune humanized immunodeficient mice (62). As discussed in this article, IL-34, but not M-CSF-1, also engages other receptors, the distribution of which in inflamed gut remains poorly characterized. At the same time, there is little knowledge about the distinction between the functional effects of such receptors and those mediated by M-CSF-1R. All these issues make the biology of IL-34 in IBD complex and far from being clearly defined. The future work will provide the necessary insight into whether IL-34 can be exploited as a therapeutic target for attenuating the detrimental immune and fibrotic responses in patients with IBD.

## Authors Contributions

GM conceptualization, manuscript preparation, supervision; EF, ET, CM literature search, writing initial draft; IM manuscript preparation, critical revision. All authors contributed to the article and approved the submitted version

## Conflict of Interest

GM has served as an advisory board member for ABBVIE. IM has served as a speaker for Janssen.

The remaining authors declare that the research was conducted in the absence of any commercial or financial relationships that could be construed as a potential conflict of interest.

## Publisher’s Note

All claims expressed in this article are solely those of the authors and do not necessarily represent those of their affiliated organizations, or those of the publisher, the editors and the reviewers. Any product that may be evaluated in this article, or claim that may be made by its manufacturer, is not guaranteed or endorsed by the publisher.

## References

[1] Torres J Mehandru S Colombel JF Peyrin-Biroulet L. Crohn's Disease. Lancet (2017) 389(10080):1741–55. doi: 10.1016/S0140-6736(16)31711-1 27914655

[2] Ungaro R Mehandru S Allen PB Peyrin-Biroulet L Colombel JF. Ulcerative Colitis. Lancet (2017) 389(10080):1756–70. doi: 10.1016/S0140-6736(16)32126-2 PMC6487890 27914657

[3] Abraham C Cho JH. Inflammatory Bowel Disease. N Engl J Med (2009) 361(21):2066–78. doi: 10.1056/NEJMra0804647 PMC3491806 19923578

[4] Macdonald TT Monteleone G. Immunity, Inflammation, and Allergy in the Gut. Science (2005) 307(5717):1920–5. doi: 10.1126/science.1106442 15790845

[5] Digby-Bell JL Atreya R Monteleone G Powell N. Interrogating Host Immunity to Predict Treatment Response in Inflammatory Bowel Disease. Nat Rev Gastroenterol Hepatol (2019) 17:9–20. doi: 10.1038/s41575-019-0228-5 31767987

[6] Zundler S Becker E Schulze LL Neurath MF. Immune Cell Trafficking and Retention in Inflammatory Bowel Disease: Mechanistic Insights and Therapeutic Advances. Gut (2019) 68(9):1688–700. doi: 10.1136/gutjnl-2018-317977 31127023

[7] Strober W Fuss IJ. Proinflammatory Cytokines in the Pathogenesis of Inflammatory Bowel Diseases. Gastroenterology (2011) 140(6):1756–67. doi: 10.1053/j.gastro.2011.02.016 PMC3773507 21530742

[8] Targan SR Hanauer SB van Deventer SJ Mayer L Present DH Braakman T. A Short-Term Study of Chimeric Monoclonal Antibody Ca2 to Tumor Necrosis Factor Alpha for Crohn's Disease. Crohn's Disease Ca2 Study Group. N Engl J Med (1997) 337(15):1029–35. doi: 10.1056/NEJM199710093371502 9321530

[9] Sandborn WJ Gasink C Gao LL Blank MA Johanns J Guzzo C. Ustekinumab Induction and Maintenance Therapy in Refractory Crohn's Disease. N Engl J Med (2012) 367(16):1519–28. doi: 10.1056/NEJMoa1203572 23075178

[10] Marafini I Sedda S Dinallo V Monteleone G. Inflammatory Cytokines: From Discoveries to Therapies in Ibd. Expert Opin Biol Ther (2019) 19(11):1207–17. doi: 10.1080/14712598.2019.1652267 31373244

[11] Dalal SR Cohen RD. What to Do When Biologic Agents Are Not Working in Inflammatory Bowel Disease Patients. Gastroenterol Hepatol (2015) 11(10):657–65. PMC4849518 27330493

[12] Lin H Lee E Hestir K Leo C Huang M Bosch E. Discovery of a Cytokine and Its Receptor by Functional Screening of the Extracellular Proteome. Science (2008) 320(5877):807–11. doi: 10.1126/science.1154370 18467591

[13] Baud'huin M Renault R Charrier C Riet A Moreau A Brion R. Interleukin-34 Is Expressed by Giant Cell Tumours of Bone and Plays a Key Role in Rankl-Induced Osteoclastogenesis. J Pathol (2010) 221(1):77–86. doi: 10.1002/path.2684 20191615

[14] Greter M Lelios I Pelczar P Hoeffel G Price J Leboeuf M. Stroma-Derived Interleukin-34 Controls the Development and Maintenance of Langerhans Cells and the Maintenance of Microglia. Immunity (2012) 37(6):1050–60. doi: 10.1016/j.immuni.2012.11.001 PMC4291117 23177320

[15] Wang Y Szretter KJ Vermi W Gilfillan S Rossini C Cella M. Il-34 Is a Tissue-Restricted Ligand of Csf1r Required for the Development of Langerhans Cells and Microglia. Nat Immunol (2012) 13(8):753–60. doi: 10.1038/ni.2360 PMC3941469 22729249

[16] Preisser L Miot C Le Guillou-Guillemette H Beaumont E Foucher ED Garo E. Il-34 and Macrophage Colony-Stimulating Factor Are Overexpressed in Hepatitis C Virus Fibrosis and Induce Profibrotic Macrophages That Promote Collagen Synthesis by Hepatic Stellate Cells. Hepatology (2014) 60(6):1879–90. doi: 10.1002/hep.27328 25066464

[17] Franze E Di Grazia A Sica GS Biancone L Laudisi F Monteleone G. Interleukin-34 Enhances the Tumor Promoting Function of Colorectal Cancer-Associated Fibroblasts. Cancers (2020) 12(12):3537. doi: 10.3390/cancers12123537 PMC7761053 33260828

[18] Franze E Dinallo V Laudisi F Di Grazia A Di Fusco D Colantoni A. Interleukin-34 Stimulates Gut Fibroblasts to Produce Collagen Synthesis. J Crohns Colitis (2020) 14(10):1436–45. doi: 10.1093/ecco-jcc/jjaa073 32271873

[19] Franze E Dinallo V Rizzo A Di Giovangiulio M Bevivino G Stolfi C. Interleukin-34 Sustains Pro-Tumorigenic Signals in Colon Cancer Tissue. Oncotarget (2018) 9(3):3432–45. doi: 10.18632/oncotarget.23289 PMC5790474 29423057

[20] Franze E Laudisi F Di Grazia A Maronek M Bellato V Sica G. Macrophages Produce and Functionally Respond to Interleukin-34 in Colon Cancer. Cell Death Discov (2020) 6(1):117. doi: 10.1038/s41420-020-00350-7 33298879 PMC7644720

[21] Franze E Marafini I De Simone V Monteleone I Caprioli F Colantoni A. Interleukin-34 Induces Cc-Chemokine Ligand 20 in Gut Epithelial Cells. J Crohns Colitis (2016) 10(1):87–94. doi: 10.1093/ecco-jcc/jjv181 26449789

[22] Franze E Marafini I Troncone E Salvatori S Monteleone G. Interleukin-34 Promotes Tumorigenic Signals for Colon Cancer Cells. Cell Death Discov (2021) 7(1):245. doi: 10.1038/s41420-021-00636-4 34535634 PMC8448832

[23] Franze E Monteleone I Cupi ML Mancia P Caprioli F Marafini I. Interleukin-34 Sustains Inflammatory Pathways in the Gut. Clin Sci (2015) 129(3):271–80. doi: 10.1042/CS20150132 25800277

[24] Baghdadi M Umeyama Y Hama N Kobayashi T Han N Wada H. Interleukin-34, a Comprehensive Review. J Leukoc Biol (2018) 104(5):931–51. doi: 10.1002/JLB.MR1117-457R 30066957

[25] Freuchet A Salama A Remy S Guillonneau C Anegon I. Il-34 and Csf-1, Deciphering Similarities and Differences at Steady State and in Diseases. J Leukoc Biol (2021) 110(4):771–96. doi: 10.1002/JLB.3RU1120-773R 33600012

[26] Nakamichi Y Udagawa N Takahashi N. Il-34 and Csf-1: Similarities and Differences. J Bone Miner Metab (2013) 31(5):486–95. doi: 10.1007/s00774-013-0476-3 23740288

[27] Felix J Elegheert J Gutsche I Shkumatov AV Wen Y Bracke N. Human Il-34 and Csf-1 Establish Structurally Similar Extracellular Assemblies With Their Common Hematopoietic Receptor. Structure (2013) 21(4):528–39. doi: 10.1016/j.str.2013.01.018 23478061

[28] Ma X Lin WY Chen Y Stawicki S Mukhyala K Wu Y. Structural Basis for the Dual Recognition of Helical Cytokines Il-34 and Csf-1 by Csf-1r. Structure (2012) 20(4):676–87. doi: 10.1016/j.str.2012.02.010 22483114

[29] Liu H Leo C Chen X Wong BR Williams LT Lin H. The Mechanism of Shared But Distinct Csf-1r Signaling by the Non-Homologous Cytokines Il-34 and Csf-1. Biochim Biophys Acta (2012) 1824(7):938–45. doi: 10.1016/j.bbapap.2012.04.012 PMC3372767 22579672

[30] Chihara T Suzu S Hassan R Chutiwitoonchai N Hiyoshi M Motoyoshi K. Il-34 and M-Csf Share the Receptor Fms But Are Not Identical in Biological Activity and Signal Activation. Cell Death Differ (2010) 17(12):1917–27. doi: 10.1038/cdd.2010.60 20489731

[31] Boulakirba S Pfeifer A Mhaidly R Obba S Goulard M Schmitt T. Il-34 and Csf-1 Display an Equivalent Macrophage Differentiation Ability But a Different Polarization Potential. Sci Rep (2018) 8(1):256. doi: 10.1038/s41598-017-18433-4 29321503 PMC5762882

[32] Wei S Nandi S Chitu V Yeung YG Yu W Huang M. Functional Overlap But Differential Expression of Csf-1 and Il-34 in Their Csf-1 Receptor-Mediated Regulation of Myeloid Cells. J Leukoc Biol (2010) 88(3):495–505. doi: 10.1189/jlb.1209822 20504948 PMC2924605

[33] Bezie S Freuchet A Serazin C Salama A Vimond N Anegon I. Il-34 Actions on Foxp3(+) Tregs and Cd14(+) Monocytes Control Human Graft Rejection. Front Immunol (2020) 11:1496. doi: 10.3389/fimmu.2020.01496 32849510 PMC7431608

[34] Foucher ED Blanchard S Preisser L Garo E Ifrah N Guardiola P. Il-34 Induces the Differentiation of Human Monocytes Into Immunosuppressive Macrophages. Antagonistic Effects of Gm-Csf and Ifngamma. PloS One (2013) 8(2):e56045. doi: 10.1371/journal.pone.0056045 23409120 PMC3568045

[35] Wiktor-Jedrzejczak WW Ahmed A Szczylik C Skelly RR. Hematological Characterization of Congenital Osteopetrosis in Op/Op Mouse. Possible Mechanism for Abnormal Macrophage Differentiation. J Exp Med (1982) 156(5):1516–27. doi: 10.1084/jem.156.5.1516 PMC2186832 7130905

[36] Yoshida H Hayashi S Kunisada T Ogawa M Nishikawa S Okamura H. The Murine Mutation Osteopetrosis Is in the Coding Region of the Macrophage Colony Stimulating Factor Gene. Nature (1990) 345(6274):442–4. doi: 10.1038/345442a0 2188141

[37] Wiktor-Jedrzejczak W Bartocci A Ferrante AW Jr. Ahmed-Ansari A Sell KW Pollard JW. Total Absence of Colony-Stimulating Factor 1 in the Macrophage-Deficient Osteopetrotic (Op/Op) Mouse. Proc Natl Acad Sci USA (1990) 87(12):4828–32. doi: 10.1073/pnas.87.12.4828 PMC54211 2191302

[38] Kana V Desland FA Casanova-Acebes M Ayata P Badimon A Nabel E. Csf-1 Controls Cerebellar Microglia and Is Required for Motor Function and Social Interaction. J Exp Med (2019) 216(10):2265–81. doi: 10.1084/jem.20182037 PMC6781012 31350310

[39] Huynh D Akcora D Malaterre J Chan CK Dai XM Bertoncello I. Csf-1 Receptor-Dependent Colon Development, Homeostasis and Inflammatory Stress Response. PloS One (2013) 8(2):e56951. doi: 10.1371/journal.pone.0056951 23451116 PMC3579891

[40] Ramsay RG Micallef SJ Williams B Lightowler S Vincan E Heath JK. Colony-Stimulating Factor-1 Promotes Clonogenic Growth of Normal Murine Colonic Crypt Epithelial Cells in Vitro. J Interferon Cytokine Res (2004) 24(7):416–27. doi: 10.1089/1079990041535638 15296653

[41] Sehgal A Donaldson DS Pridans C Sauter KA Hume DA Mabbott NA. The Role of Csf1r-Dependent Macrophages in Control of the Intestinal Stem-Cell Niche. Nat Commun (2018) 9(1):1272. doi: 10.1038/s41467-018-03638-6 29593242 PMC5871851

[42] Huynh D Dai XM Nandi S Lightowler S Trivett M Chan CK. Colony Stimulating Factor-1 Dependence of Paneth Cell Development in the Mouse Small Intestine. Gastroenterology (2009) 137(1):136–44, 44 e1-3. doi: 10.1053/j.gastro.2009.03.004 19303020 PMC2706482

[43] Lin W Xu D Austin CD Caplazi P Senger K Sun Y. Function of Csf1 and Il34 in Macrophage Homeostasis, Inflammation, and Cancer. Front Immunol (2019) 10:2019. doi: 10.3389/fimmu.2019.02019 31552020 PMC6736990

[44] Stadler M Pudelko K Biermeier A Walterskirchen N Gaigneaux A Weindorfer C. Stromal Fibroblasts Shape the Myeloid Phenotype in Normal Colon and Colorectal Cancer and Induce Cd163 and Ccl2 Expression in Macrophages. Cancer Lett (2021) 520:184–200. doi: 10.1016/j.canlet.2021.07.006 34256095

[45] Gonzalez-Dominguez E Samaniego R Flores-Sevilla JL Campos-Campos SF Gomez-Campos G Salas A. Cd163l1 and Clec5a Discriminate Subsets of Human Resident and Inflammatory Macrophages in Vivo. J Leukoc Biol (2015) 98(4):453–66. doi: 10.1189/jlb.3HI1114-531R 25877931

[46] Bain CC Schridde A. Origin, Differentiation, and Function of Intestinal Macrophages. Front Immunol (2018) 9:2733. doi: 10.3389/fimmu.2018.02733 30538701 PMC6277706

[47] Massimino L Lamparelli LA Houshyar Y D’Alessio S Peyrin-Biroulet L Vetrano S. The Inflammatory Bowel Disease Transcriptome and Metatranscriptome Meta-Analysis (IBD TaMMA) Framework. Nat Comput Sci (2021) 1:511–5. doi: 10.1038/s43588-021-00114-y PMC10766544 38217242

[48] Manthey CL Moore BA Chen Y Loza MJ Yao X Liu H. The Csf-1-Receptor Inhibitor, Jnj-40346527 (Prv-6527), Reduced Inflammatory Macrophage Recruitment to the Intestinal Mucosa and Suppressed Murine T Cell Mediated Colitis. PloS One (2019) 14(11):e0223918. doi: 10.1371/journal.pone.0223918 31710624 PMC6844469

[49] Zwicker S Martinez GL Bosma M Gerling M Clark R Majster M. Interleukin 34: A New Modulator of Human and Experimental Inflammatory Bowel Disease. Clin Sci (2015) 129(3):281–90. doi: 10.1042/CS20150176 PMC4557398 25896238

[50] Kontoyiannis D Pasparakis M Pizarro TT Cominelli F Kollias G. Impaired on/Off Regulation of Tnf Biosynthesis in Mice Lacking Tnf Au-Rich Elements: Implications for Joint and Gut-Associated Immunopathologies. Immunity (1999) 10(3):387–98. doi: 10.1016/S1074-7613(00)80038-2 10204494

[51] Guillonneau C Bezie S Anegon I. Immunoregulatory Properties of the Cytokine Il-34. Cell Mol Life Sci (2017) 74(14):2569–86. doi: 10.1007/s00018-017-2482-4 PMC11107603 28258292

[52] Ciccia F Alessandro R Rodolico V Guggino G Raimondo S Guarnotta C. Il-34 Is Overexpressed in the Inflamed Salivary Glands of Patients With Sjogren's Syndrome and Is Associated With the Local Expansion of Pro-Inflammatory Cd14(Bright)Cd16+ Monocytes. Rheumatology (Oxford England) (2013) 52(6):1009–17. doi: 10.1093/rheumatology/kes435 23392590

[53] Zhao Z Pan G Tang C Li Z Zheng D Wei X. Il-34 Inhibits Acute Rejection of Rat Liver Transplantation by Inducing Kupffer Cell M2 Polarization. Transplantation (2018) 102(6):e265–74. doi: 10.1097/TP.0000000000002194 29570162

[54] Foucher ED Blanchard S Preisser L Descamps P Ifrah N Delneste Y. Il-34- and M-Csf-Induced Macrophages Switch Memory T Cells Into Th17 Cells *Via* Membrane Il-1alpha. Eur J Immunol (2015) 45(4):1092–102. doi: 10.1002/eji.201444606 25545357

[55] Troncone E Marafini I Pallone F Monteleone G. Th17 Cytokines in Inflammatory Bowel Diseases: Discerning the Good From the Bad. Int Rev Immunol (2013) 32(5-6):526–33. doi: 10.3109/08830185.2013.823421 24041379

[56] Zorzi F Monteleone I Sarra M Calabrese E Marafini I Cretella M. Distinct Profiles of Effector Cytokines Mark the Different Phases of Crohn's Disease. PloS One (2013) 8(1):e54562. doi: 10.1371/journal.pone.0054562 23349929 PMC3547873

[57] Muller WA. Leukocyte-Endothelial-Cell Interactions in Leukocyte Transmigration and the Inflammatory Response. Trends Immunol (2003) 24(6):327–34. doi: 10.1016/s1471-4906(03)00117-0 12810109

[58] Kwon JH Keates S Bassani L Mayer LF Keates AC. Colonic Epithelial Cells Are a Major Site of Macrophage Inflammatory Protein 3alpha (Mip-3alpha) Production in Normal Colon and Inflammatory Bowel Disease. Gut (2002) 51(6):818–26. doi: 10.1136/gut.51.6.818 PMC1773480 12427784

[59] Rieder F Fiocchi C. Intestinal Fibrosis in Inflammatory Bowel Disease: Progress in Basic and Clinical Science. Curr Opin Gastroenterol (2008) 24(4):462–8. doi: 10.1097/MOG.0b013e3282ff8b36 18622160

[60] Zorzi F Calabrese E Monteleone G. Pathogenic Aspects and Therapeutic Avenues of Intestinal Fibrosis in Crohn's Disease. Clin Sci (2015) 129(12):1107–13. doi: 10.1042/CS20150472 26494636

[61] Rieder F Fiocchi C. Intestinal Fibrosis in Ibd–a Dynamic, Multifactorial Process. Nat Rev Gastroenterol Hepatol (2009) 6(4):228–35. doi: 10.1038/nrgastro.2009.31 19347014

[62] Wynn TA. Cellular and Molecular Mechanisms of Fibrosis. J Pathol (2008) 214(2):199–210. doi: 10.1002/path.2277 18161745 PMC2693329

[63] Rieder F Fiocchi C. Intestinal Fibrosis in Inflammatory Bowel Disease - Current Knowledge and Future Perspectives. J Crohns Colitis (2008) 2(4):279–90. doi: 10.1016/j.crohns.2008.05.009 21172225

[64] Fiocchi C Lund PK. Themes in Fibrosis and Gastrointestinal Inflammation. Am J Physiol Gastrointest Liver Physiol (2011) 300(5):G677–83. doi: 10.1152/ajpgi.00104.2011 PMC3094134 21415411

[65] Kendall RT Feghali-Bostwick CA. Fibroblasts in Fibrosis: Novel Roles and Mediators. Front Pharmacol (2014) 5:123. doi: 10.3389/fphar.2014.00123 24904424 PMC4034148

[66] Lawrance IC Rogler G Bamias G Breynaert C Florholmen J Pellino G. Cellular and Molecular Mediators of Intestinal Fibrosis. J Crohns Colitis (2017) 11(12):1491–503. doi: 10.1016/j.crohns.2014.09.008 PMC5885809 25306501

[67] Fichtner-Feigl S Young CA Kitani A Geissler EK Schlitt HJ Strober W. Il-13 Signaling *Via* Il-13r Alpha2 Induces Major Downstream Fibrogenic Factors Mediating Fibrosis in Chronic Tnbs Colitis. Gastroenterology (2008) 135(6):2003–13, 13 e1-7. doi: 10.1053/j.gastro.2008.08.055 18938165

[68] Johnson LA Rodansky ES Haak AJ Larsen SD Neubig RR Higgins PD. Novel Rho/Mrtf/Srf Inhibitors Block Matrix-Stiffness and Tgf-Beta-Induced Fibrogenesis in Human Colonic Myofibroblasts. Inflamm Bowel Dis (2014) 20(1):154–65. doi: 10.1097/01.MIB.0000437615.98881.31 PMC4893808 24280883

[69] Li G Ren J Hu Q Deng Y Chen G Guo K. Oral Pirfenidone Protects Against Fibrosis by Inhibiting Fibroblast Proliferation and Tgf-Beta Signaling in a Murine Colitis Model. Biochem Pharmacol (2016) 117:57–67. doi: 10.1016/j.bcp.2016.08.002 27498142

[70] Imai J Hozumi K Sumiyoshi H Yazawa M Hirano K Abe J. Anti-Fibrotic Effects of a Novel Small Compound on the Regulation of Cytokine Production in a Mouse Model of Colorectal Fibrosis. Biochem Biophys Res Commun (2015) 468(4):554–60. doi: 10.1016/j.bbrc.2015.10.123 26603932

[71] Speca S Rousseaux C Dubuquoy C Rieder F Vetuschi A Sferra R. Novel Ppargamma Modulator Ged-0507-34 Levo Ameliorates Inflammation-Driven Intestinal Fibrosis. Inflamm Bowel Dis (2016) 22(2):279–92. doi: 10.1097/MIB.0000000000000618 PMC4718865 26535766

[72] Zhou J Sun X Zhang J Yang Y Chen D Cao J. Il-34 Regulates Il-6 and Il-8 Production in Human Lung Fibroblasts *Via* Mapk, Pi3k-Akt, Jak and Nf-Kappab Signaling Pathways. Int Immunopharmacol (2018) 61:119–25. doi: 10.1016/j.intimp.2018.05.023 29857241

[73] Wang B Ma Z Wang M Sun X Tang Y Li M. Il-34 Upregulated Th17 Production Through Increased Il-6 Expression by Rheumatoid Fibroblast-Like Synoviocytes. Mediators Inflamm (2017) 2017:1567120. doi: 10.1155/2017/1567120 28659662 PMC5474253

[74] Li X Lei Y Gao Z Wu G Gao W Xia L. Il-34 Affects Fibroblast-Like Synoviocyte Proliferation, Apoptosis and Function by Regulating Il-17. Sci Rep (2021) 11(1):16378. doi: 10.1038/s41598-021-95839-1 34385542 PMC8361173

[75] Elkhider A Wei J Al-Azab M Tang Y Walana W Li W. Il-34 Modulates Rheumatoid Synovial Fibroblasts Proliferation and Migration *Via* Erk/Akt Signalling Pathway. Clin Exp Rheumatol (2020) 38(3):479–87. 31498070

[76] Kuzumi A Yoshizaki A Toyama S Fukasawa T Ebata S Nakamura K. Serum Interleukin-34 Levels in Patients With Systemic Sclerosis: Clinical Association With Interstitial Lung Disease. J Dermatol (2018) 45(10):1216–20. doi: 10.1111/1346-8138.14538 30004593

[77] Shoji H Yoshio S Mano Y Kumagai E Sugiyama M Korenaga M. Interleukin-34 as a Fibroblast-Derived Marker of Liver Fibrosis in Patients With Non-Alcoholic Fatty Liver Disease. Sci Rep (2016) 6:28814. doi: 10.1038/srep28814 27363523 PMC4929441

